# Dependence of the Magnetization Process on the Thickness of Fe_70_Pd_30_ Nanostructured Thin Film

**DOI:** 10.3390/ma13245788

**Published:** 2020-12-18

**Authors:** Mohamed Salaheldeen, Ahmed Mohamed Abu-Dief, Lucía Martínez-Goyeneche, Seraj Omar Alzahrani, Fatmah Alkhatib, Pablo Álvarez-Alonso, Jesús Ángel Blanco

**Affiliations:** 1 Physics Department, Faculty of Science, Sohag University, Sohag 82524, Egypt; 2 Departamento de Física, Universidad de Oviedo, C/Calvo Sotelo 18, 33007 Oviedo, Spain; martinezglucia@uniovi.es (L.M.-G.); jabr@uniovi.es (J.Á.B.); 3 Chemistry Department, College of Science, Taibah University, Madinah P.O. Box 344, Saudi Arabia; amamohammed@taibahu.edu.sa (A.M.A.-D.); szahrani@taibahu.edu.sa (S.O.A.); 4 Chemistry Department, Faculty of Science, Sohag University, Sohag 82534, Egypt; 5 Chemistry Department, Faculty of Applied Science, Umm Al-Qura University, Makkah P.O. Box 715, Saudi Arabia; fmkhatib@uqu.edu.sa

**Keywords:** nanoporous alumina templates, antidot arrays, Kerr effect, Curie temperature, Fe_70_Pd_30_ shape-memory alloy

## Abstract

Fe–Pd magnetic shape-memory alloys are of major importance for microsystem applications due to their magnetically driven large reversible strains under moderate stresses. In this context, we focus on the synthesis of nanostructured Fe_70_Pd_30_ shape-memory alloy antidot array thin films with different layer thicknesses in the range from 20 nm to 80 nm, deposited onto nanostructured alumina membranes. A significant change in the magnetization process of nanostructured samples was detected by varying the layer thickness. The in-plane coercivity for the antidot array samples increased with decreasing layer thickness, whereas for non-patterned films the coercive field decreased. Anomalous coercivity dependence with temperature was detected for thinner antidot array samples, observing a critical temperature at which the in-plane coercivity behavior changed. A significant reduction in the Curie temperature for antidot samples with thinner layer thicknesses was observed. We attribute these effects to complex magnetization reversal processes and the three-dimensional magnetization profile induced by the nanoholes. These findings could be of major interest in the development of novel magnetic sensors and thermo-magnetic recording patterned media based on template-assisted deposition techniques.

## 1. Introduction

Fe–Pd alloy thin films have attracted interest due to their various functional properties: strong effective uniaxial magnetic anisotropy, large saturation magnetization, high magneto-optic Kerr rotation behavior [1], magnetic behavior [2], biocompatibility [3,4], mechanical properties [5] and wettability properties [6]. In particular, Fe–Pd alloys containing about 30 at.% Pd are promising magnetic shape-memory alloys, since they show a ferromagnetic character at room temperature [7,8,9]. These shape-memory alloys are smart materials that, due to their potential applications, have attracted a great deal of attention from materials researchers—especially in wireless actuated micro/nanoelectromechanical systems, magneto-mechanical actuators and sensors [9,10]. The shape-memory effect is based on the reorientation of the twin variants of the martensite phase associated with Fe–Pd alloys [11] and can be controlled by temperature and stress. However, the magnetic field control of the shape-memory effect has been put forward as a principle to design a new type of microstructure actuator in which the reorganization of twins can be accomplished by an external applied magnetic field [7]. The combination of magnetic and martensitic order makes this metallic multiferroic material a promising candidate for applications in micro- and nanodevices [10]. Recently, Fe–Pd alloy nanostructure arrays based on self-assembling techniques have shown a strong potential for energy-assisted recording on nanoscale magnetic media, ultrafast spintronic technology and nano-actuators in biomedical applications [11,12,13,14]. For such applications, tailoring the direction and strength of the magnetic anisotropy, especially for the thermal stability and switching reliability of magnetic bits, is a crucial prerequisite [15]. In this regard, the existence of ordered arrays of nanoholes (i.e., antidot arrays) induces a demagnetization field distribution around the holes that can completely alter the magnetic properties of a non-patterned thin film, such as its switching field, magnetization reversal mechanism and intrinsic magnetic anisotropy [16,17,18,19,20].

The effect of geometrical parameters, especially the thickness, on the magnetic properties of Fe–Pd alloys based on a hexagonal antidot array thin film has not been investigated before. Moreover, the thermal-magnetic behavior of these nanostructured alloys with different geometrical parameters is not well understood. Furthermore, the behavior of Fe–Pd shape-memory alloys deposited as antidot nanostructures has been barely studied [13,21,22], and no analysis of the effect of geometric parameters has been conducted. Therefore, our aim is to contribute to the knowledge of the dependence of the nanostructural geometry on the magnetic properties of Fe_70_Pd_30_ thin films.

In the current investigation, we highlight our recent developments in the fabrication and characterization of nanostructured Fe_70_Pd_30_ alloy thin films with different layer thicknesses. The magnetic properties of both nanostructured and corresponding non-patterned films were investigated. Anomalous magnetic behaviors for nanostructured samples with layer thickness *t* ≤ 40 nm were observed. Critical temperatures of 100 K and 60 K for antidot thin films with respective layer thicknesses of 20 nm and 40 nm were found, for which the coercivity field reached a maximum and decreased with a further reduction of the temperature. In addition, a noticeable decrease in the Curie temperature from 670 K for the non-patterned thin film to 555 K in the case of nanostructured samples with a layer thickness of 20 nm was detected. The current results may represent significant advances in the study of novel nanostructured magnetic shape-memory alloys in template-assisted deposition techniques, with large potential in applications like thermo-magnetic recording and novel magnetic sensors.

## 2. Materials and Methods

### 2.1. Fabrication of Fe–Pd Antidot Thin Films

Nanoporous anodic alumina templates (NPAATs) with a square surface of 4 cm^2^, thickness of 500 μm, inter-hole distance *P* = 107 ± 3 nm, and hole diameter *D* = 80 ± 5 nm were fabricated via two-step mild anodization of high purity (99.999%) Al foils, which were electropolished with a mixture of H_3_PO_4_ and H_2_SO_4_ to improve the surface smoothness [23]. After cleaning and electropolishing at 50 V in perchloric acid and ethanol solution (1:3 vol., 9 °C) for 8 min, the Al foils were subjected to a two-step electrochemical anodization, as described elsewhere [23,24]. During the second anodization step, which lasted 6 h, the nanopores grew following the prepatterned engineering of a highly self-ordered hexagonal symmetry produced in the first anodization process. Finally, nanoporous alumina samples were chemically etched in 6 wt.% orthophosphoric acid at 40 °C for 48 min.

Hexagonally ordered antidot arrays of Fe_70_Pd_30_ alloy thin films formed by highly pure metal pieces of Fe (Goodfellow Limited, Cambridge, UK, 99.9% purity) and Pd (Ventron GMBH, Dortmund, Germany, 99.99% purity) were deposited on the NPAAT substrates by an ultra-high vacuum thermal evaporator (Edwards E306A, Manor Royal, Crawley, West Sussex, UK) at pressures below 10^−7^ mbar, as described in [25,26,27], with a film thickness in the range of 20 nm ≤ *t* ≤ 80 nm (in steps of 20 nm). Continuous Fe–Pd alloy thin films (CTFs) were also deposited on a glass substrate at the same time as the antidot samples for comparison. Magnetically focused electron beams with 3.1 kV for Fe (crucible 1) and 4.5 kV for Pd (crucible 2); and electric energy 2.7 kWh for (Fe) and 2.3 kWh for (Pd) were used to evaporate the Fe and Pd targets, respectively. The evaporated target metals were both deposited on the top surface of the NPAAT substrates, which acted as templates to obtain the nanostructured array films [26,27]. A crystal quartz thickness monitor was used to control the film thickness during the evaporation process, where the layer thickness increased from 20 to 80 nm. The distance between the evaporation source and crystal quartz was kept constant at about 18 cm, and the deposition rate was around 0.1–0.15 nm/s.

### 2.2. Morphological Characterization and Composition

The morphological characterization of antidot and continuous samples was performed using scanning electron microscopy (SEM) (JEOL-6610LV, JEOL Ltd., Tokyo, Japan) equipped with an energy-dispersive spectroscopy (EDS) system. High-resolution transmission electron microscopy (HR-TEM) (JEM 2100, JEOL, JEOL Ltd., Tokyo, Japan) operating at 200 kV was employed to obtain different magnification images of the Fe–Pd continuous thin films. For this purpose, the non-patterned sample was manually milled, and the obtained powder was dispersed in ethanol by ultrasonication and localized in a carbon grid. Selected area electron diffraction (SAED) spectra were then collected. The chemical composition of the antidots and their corresponding continuous thin films were determined via the crystal quartz control at the end of the film deposition, used to calculate the Fe:Pd ratio in the alloy [28]. To confirm the composition of the Fe–Pd alloy, subsequent analysis was performed by energy-dispersive X-ray spectroscopy (EDX, JEOL Ltd., Tokyo, Japan), as plotted in Table 1.

### 2.3. Magnetic Properties Characterization

The magnetic properties of the Fe_70_Pd_30_ shape-memory alloy thin films and their corresponding CTFs were investigated from the hysteresis loops measured at room temperature on the surface of the samples via the transversal magneto-optic Kerr effect (T-MOKE), with the applied magnetic field reaching values of up to 0.25 T. Furthermore, a vibrating-sample magnetometer was employed in the temperature range between 50 K and 750 K and an applied magnetic field of up to 2 T was directed along the plane of the film.

## 3. Results

### 3.1. Morphology and Composition

Analysis of the EDS spectra with an acceleration voltage of 20 kV of the CTF samples corresponding to different layer thicknesses provided a 67:33 (*t* = 20 nm), 69:31 (*t* = 40 nm), 70:30 (*t* = 60 nm) and 68:32 (*t* = 80 nm) Fe:Pd ratio, as summarized in Table 1. The uncertainties were estimated as the standard deviation (four different points on the surface were taken for each specimen); the relatively low values of the standard deviation assured the homogeneity of the samples. The small variations in the final chemical composition of the samples were most likely due to the procedures during the film deposition to fix the deposition rate of Fe and Pd elements with its layer thickness, as reported in [28].

Regarding the homogeneity of the samples, the non-patterned samples showed good homogeneity, checked by both the reflectivity change in the whole of the film with transversal MOKE and EDS analysis. However, the nanostructured samples showed a high variation of the reflectivity due to the high surface roughness of the nanoporous alumina membrane. Despite this, the continuous and nanopatterned samples were fabricated in the zone of the evaporator system that guarantees the best homogeneity.

**Figure 1:**
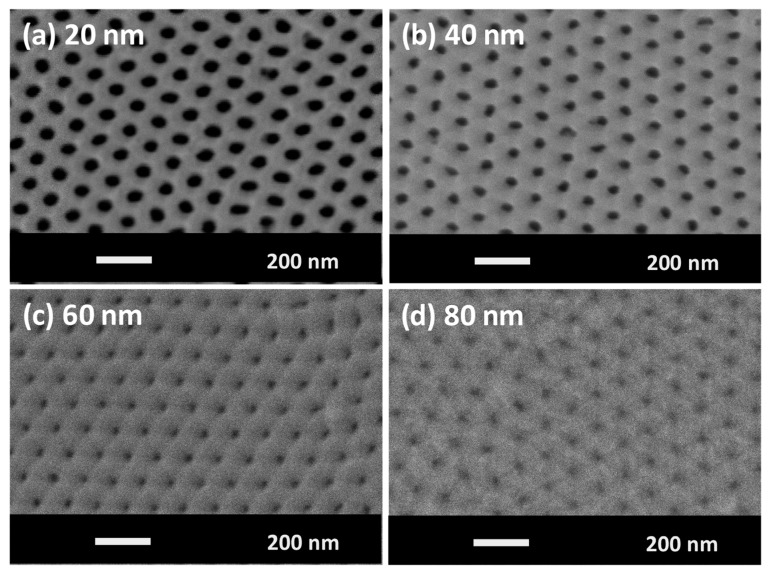
(**a**–**d**) SEM images of Fe_70_Pd_30_ nanostructured film samples with different layer thicknesses: (**a**) 20 nm, (**b**) 40 nm, (**c**) 60 nm and (**d**) 80 nm.

Figure 1a–d shows SEM images of sample surfaces of Fe–Pd alloy antidot arrays with the same hexagonal symmetry and lattice parameter starting values (*D*, *P*), but with varying thickness layers. It is worth noting that as the film thickness increased, the apparent diameter of the nanoholes decreased due to the deposition of magnetic material at the top of the hole, until it was totally closed off in the 80 nm thick antidot sample (Figure 1d). In contrast, both the edge-to-edge distance (*W*) and the magnetic surface cover ratio (*C*) increased. Table 2 summarizes the apparent geometrical parameters of the antidot array samples with different layer thicknesses.

**Figure 2:**
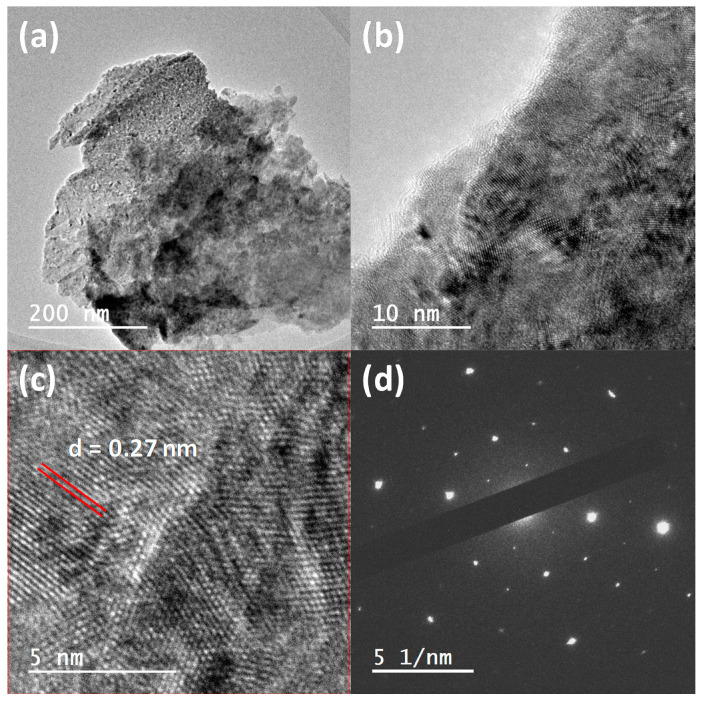
(**a**–**c**) HR-TEM images of Fe–Pd continuous thin film with a 20 nm layer thickness taken at different magnifications. Regions with different directions of the crystallographic planes are shown in (**b**,**c**). (**d**) The SAED image along a direction near a two-fold symmetry axis (see text).

Figure 2 describes the characteristic microstructure of the samples studied by HR-TEM. Defined regions with a clear periodicity of the atoms in a different direction of the crystallographic planes evidence that the film had a crystal structure, despite having been deposited on a glass substrate. The size of the areas with a well-defined crystallographic orientation was approximately 5 nm. Moreover, the presence of clear spots with well-defined symmetry in the electron diffraction spectrum in Figure 2d demonstrates the crystalline nature of the deposited films, as was observed in [3,5,31,32]. It is worth noting that Fe_x_Pd_100−x_ alloys changed crystal structure depending on the composition around *x* = 70. In fact, for *x* = 65 they adopted a face-centered cubic crystal structure, while for *x* = 75 the material was arranged in a body-centered cubic crystal structure. The observed interplanar distance 0.27 nm is larger than the distances corresponding to both cubic crystal structures. For this reason, one attempt was made considering a L1_0_ crystal structure (the space group of this crystal type-structure is P4/mmm, i.e., a simple tetragonal crystal structure) leading to a 3% difference between the cell parameter in the basal plane and the c-direction (a = b = 3.81 Å and c = 3.70 Å) [33]. In this situation, the observed distance of 0.27 nm would correspond to the (110) Bragg reflection. Nevertheless, further investigations are needed for confirming the actual crystal structure of Fe_70_Pd_30_.

### 3.2. Magnetic Properties

**Figure 3:**
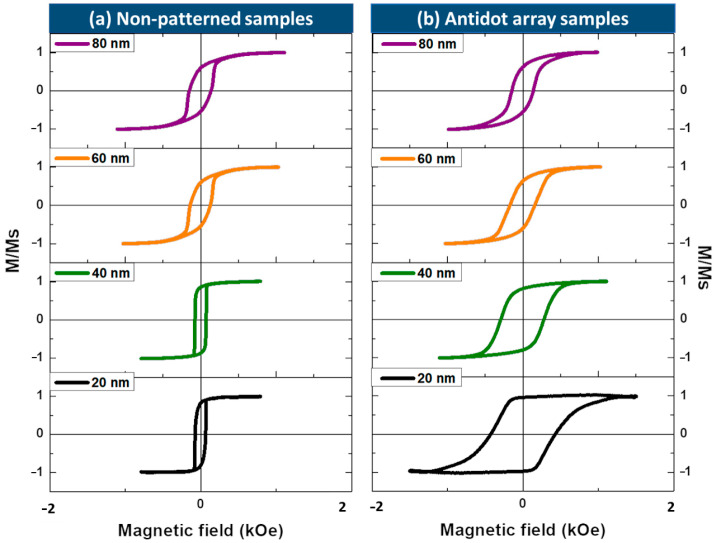
Transversal Kerr hysteresis loops for Fe_70_Pd_30_ with different thicknesses (20, 40, 60 and 80 nm) for (**a**) non-patterned thin films and (**b**) antidot array thin films.

**Figure 4:**
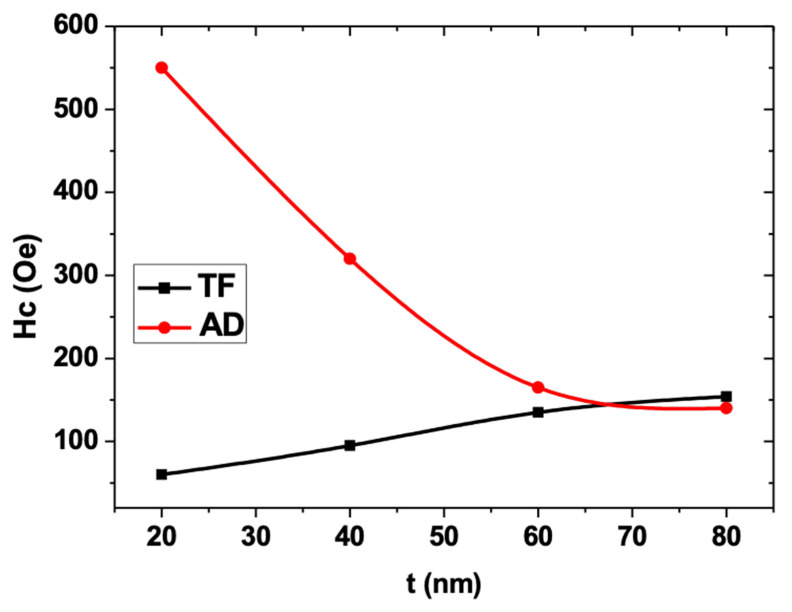
Room-temperature in-plane coercivity of the Fe_70_Pd_30_ antidot arrays and their corresponding continuous thin films as a function of layer thickness.

Figure 3a,b shows the T-MOKE hysteresis loops of the Fe_70_Pd_30_ antidot array thin films and their corresponding CTFs with a thickness range between 20 nm and 80 nm. For nanostructured and non-patterned samples, the magnetization initially laid in the in-plane direction in all thickness ranges. Whereas the loops exhibited a high remanent magnetization and coercivity, a clear variation in magnetic properties was observed between the antidot array samples and their corresponding non-patterned samples. For CTF samples, the in-plane coercivity increased slightly with increasing layer thickness, with said coercivity ranging between 65 and 155 Oe for the samples with *t* = 20 nm and 80 nm, respectively. This behavior is typical of soft magnetic materials, in which the coercivity increases with increasing film thickness because of the augmentation in surface roughness and grain size, resulting in an enlargement of the magnetic shape anisotropy for thin films of this kind [34]. This observation is in line with our experimental data, as can be appreciated in Figure 3a and Figure 4. Furthermore, an in-plane uniaxial anisotropy was observed for the continuous thin films, which could be ascribed to two different phenomena: a residual stress on the surface of the film was generated after the deposition, which is associated with the angle of incidence of the evaporation beam in the substrate, and the dispersion of the magnetic field values needed to focus the electrons used to heat the materials [28].

Nanostructuring significantly affects the shape of hysteresis loops and drastically changes the magnetic behavior of the antidot samples with layer thicknesses lower than 40 nm, as can be deduced from the comparison of the magnetic behavior of the non-patterned samples and that of the thin films of antidot arrays in Figure 3 and Figure 4, respectively. In particular, higher values of coercivity were found in the Fe_70_Pd_30_ antidot samples when compared to the values for the non-patterned thin film samples with the same layer thickness. This feature is associated with an additional pinning effect induced by the array of holes. We observed the contrary thickness-dependent behavior of *H*_C_ for the antidot samples, as illustrated in Figure 4, where it can clearly be seen that the coercivity decreases with increasing Fe_70_Pd_30_ antidot film thickness. These features of coercivity are the result of the combination of two major causes. On the one hand, as can be seen in the SEM images, the geometric parameters of the nanostructured thin films changed substantially; specifically, the differences of the CTFs decreased with increasing thickness, thereby favoring the long-distance magnetic interactions. On the other hand, an increase in film thickness led to an increase in the roughness of the film surface [26]. Hence, the roughness surface anisotropy for antidot thin films with smaller layer thicknesses (20 and 40 nm) benefits the reduction in the pinning of the magnetic domain walls ascribed to nanoholes. In this respect, domain-wall pinning predominated over the magnetic anisotropy associated with the surface roughness for antidot thin films with smaller layer thicknesses (20 and 40 nm), thus giving rise to higher coercivity values than those found in non-patterned thin films and to a reduction in coercivity with increasing thickness of the Fe_70_Pd_30_ antidot thin films. The antidot arrays with a layer thickness of 80 nm showed the typical magnetic behavior of CTFs with the same layer thickness, due to the collapse of the hexagonal symmetry, as shown in Figure 1 and Figure 3.

A magnetic anisotropy with an in-plane easy magnetization direction was obtained for the Fe_70_Pd_30_ antidot arrays (see Figure 3b). Apart from the causes outlined for the continuous thin films, nanoholes induce a strong local shape anisotropy that tends to align the magnetization parallel to their edges [19,35]. Consequently, domain wall displacements result in the in-plane magnetization process, leading to hysteresis loops with a high squareness ratio [26].

**Figure 5:**
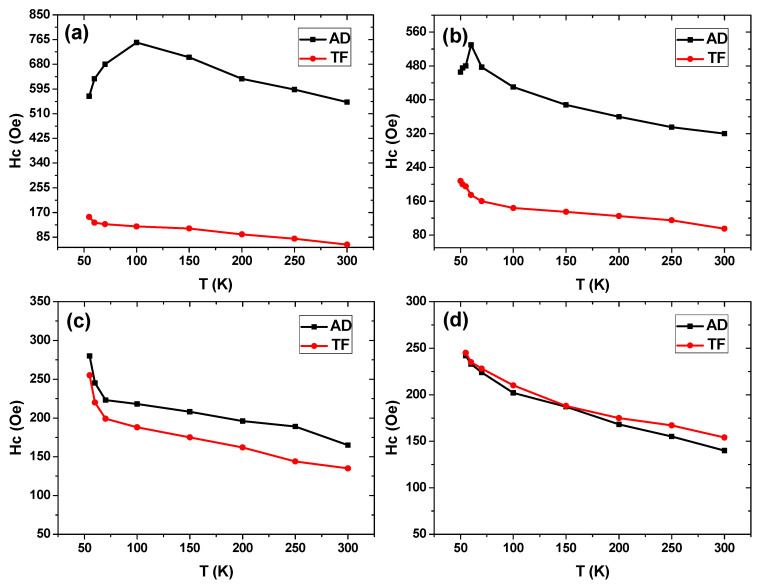
Temperature dependence of the in-plane coercivity for both nanopatterned and continuous Fe_70_Pd_30_ thin films with different layer thicknesses: (**a**) 20 nm, (**b**) 40 nm, (**c**) 60 nm and (**d**) 80 nm.

**Figure 6:**
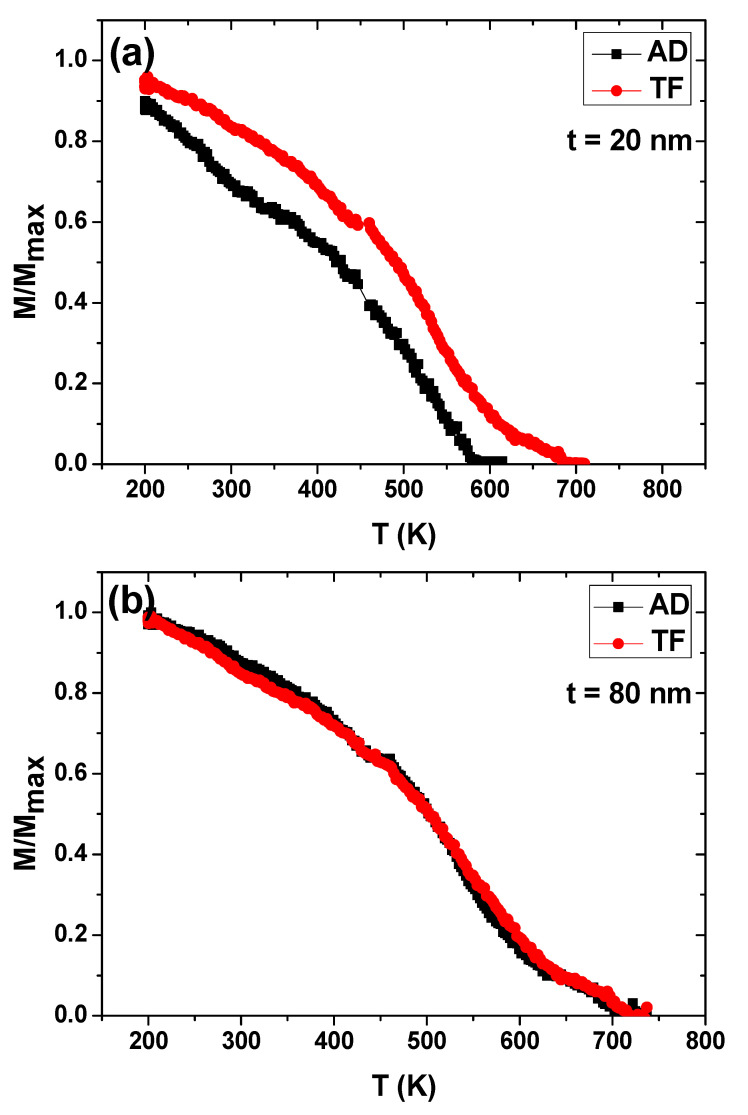
Temperature dependence of the normalized magnetization in Fe_70_Pd_30_ alloys for both continuous Fe–Pd alloy thin film (CTF) and nanostructured array samples with layer thickness of (**a**) 20 nm and (**b**) 80 nm, measured at a constant applied field of 20 kOe along the in-plane direction.

The temperature dependence of the in-plane coercivity for the nanostructured thin films and their corresponding CTF is plotted in Figure 5. Normal ferromagnetic behavior was observed for the Fe_70_Pd_30_ alloy for both the antidot and non-patterned samples. In fact, the ferromagnetic behavior of Fe_70_Pd_30_ has been observed at very low temperatures [7]. A variation in coercivity behavior was noticed when analyzing the magnetic hysteresis loops at different temperatures ranging from 300 K to 50 K for both the antidot and the corresponding non-patterned films. For continuous thin film samples with different layer thicknesses, a normal increase in coercivity with decreasing temperature was detected due to the increase in the degree of ferromagnetic ordering with decreasing temperature, as can be seen in Figure 5. For thinner antidot array samples, anomalous magnetic behavior was observed. The coercivity increased with decreasing temperature: it reached a maximum (755 Oe) at the critical temperature of 100 K for antidot samples with a layer thickness of 20 nm and then started to decrease with further increase in temperature, as can be noted in Figure 5a. A shift in the critical temperature was detected for antidot samples with a layer thickness of 40 nm, in which the maximum coercivity (530 Oe) occurred at 60 K (see Figure 6b). Liu Qing-Fang et al. observed a similar magnetic behavior in a polycrystalline Co antidot array thin film with layer thickness varying between 15 nm and 45 nm, where the in-plane coercivity reached a maximum value at 50 K, then decreased with a further reduction of the temperature. They ascribed this behavior to the complex competition between the magneto-crystalline anisotropy and the pinning effect induced by the nanostructured array of holes [36]. In addition, as they demonstrated, for the temperature range 50–300 K, the behavior of the coercivity was determined by the magneto-crystalline anisotropy, while for temperatures lower than 50 K the pinning effect had a dominant role on the coercivity behavior [36]. In our current results, for the thicker antidot array samples, a similar behavior to their non-patterned thin films was observed, as can be seen in Figure 6a,b, for which the pinning effect was reduced with increasing layer thickness, or, in other words, the behavior of coercivity was determined by the magneto-crystalline anisotropy. Thus, we believe that the strong pinning effect induced by the array of holes could play a major role in the novel coercivity behavior shown by the Fe_70_Pd_30_ nanostructured array thin film with *t* ≤ 40 nm, especially at low temperatures.

To investigate the effects of the thermal activation on nanostructured array films and CTF samples, we measured the temperature dependence of the magnetization, *M*(*T*), in the temperature interval from 200 K to 750 K. Figure 6 plots the normalized magnetization, *M*(*T*)/*M*(RT), as a function of the temperature for Fe_70_Pd_30_ non-patterned films and the corresponding nanostructured array films with *t* = 20 nm and 80 nm. We applied a high external magnetic field of around 20 kOe because of the very weak magnetic signal in the case of nanostructured samples, and followed the procedures reported in [28]. As can be seen in Figure 6, a variation in magnetic behavior was detected for antidot array samples with different antidot layer thicknesses. A sharp drop in the magnetization curve for antidot array samples with a layer thickness of 20 nm was observed, which is greater than that found in the CTF samples, because of the strong pinning effect induced by the nanoholes. Therefore, a significant reduction in the Curie temperature, *T*_C_, was observed (Figure 6a), compared to the corresponding continuous film. To obtain an accurate value of *T*_C_, a fitting of the magnetization curves plotted in Figure 6 was performed according to the method discussed in [28]. The decrease in *T*_C_ from 670 K for a non-patterned thin film to 555 K in the case of antidot array thin films is related to the confinement of the magnetic film surrounding the nanohole (smaller activation volume) compared to the non-patterned thin films. Moreover, the existence of hexagonal arrays of holes in the antidot samples resulted in a complex three-dimensional magnetization texture related to proximity effects around the holes in small finite regions [37,38,39]. The CTF and nano-patterned samples with an 80 nm layer thickness showed similar thermal-magnetic behavior due to the increase in the activation volume associated with the strong change in the nanostructure, as shown in Figure 1d and Figure 6b.

## 4. Conclusions

The magnetic behavior of nanostructured Fe_70_Pd_30_ alloy antidot arrays and their corresponding continuous thin films was investigated. The structural analysis of Fe_70_Pd_30_ alloy thin films by HR-TEM seems to suggest the L1_0_ crystal structure of the samples. A noticeable variation in the magnetic behavior of antidot array samples as a function of layer thickness and temperature was observed. The thinnest antidot array sample showed the highest in-plane coercivity due to the highest pinning effect. The coercivity increased with decreasing antidot layer thickness, while the opposite occurred for the non-patterned samples. Furthermore, a critical temperature was detected below 100 K (at which the maximum value of coercivity was obtained) where the coercivity behavior changed with temperature. A sharp reduction in the Curie temperature was evidenced for antidot samples with a thickness of 20 nm compared to the corresponding non-patterned CTF with the same thickness. Confinement of the magnetic film surrounding and between the nanoholes suggests the possibility of complex magnetic texturing and a strong pinning effect induced by the nanoholes that reduced the Curie temperature of the nanostructured system. These findings provide additional prospects to tailor the Curie temperature of hosting magnetic materials deposited on a nanoporous alumina membrane as a template. Finally, the present results could be of major interest in the development of novel magnetic shape-memory alloy sensors and thermo-magnetic recording patterned media, based on template-assisted deposition techniques.

## Figures and Tables

**Table 1:** Atomic percentage of Fe and Pd elemental composition in Fe–Pd thin films with different layer thicknesses.

Sample	S1 (20 nm)		S2 (40 nm)		S3 (60 nm)		S4 (80 nm)	
Atomic Species	Fe (at.%)	Pd (at.%)	Fe (at.%)	Pd (at.%)	Fe (at.%)	Pd (at.%)	Fe (at.%)	Pd (at.%)
Average	67 ± 2	33 ± 2	69 ± 3	31 ± 3	70 ± 3	30 ± 3	68 ± 3	32 ± 3

**Table 2:** The apparent geometrical parameters of Fe_70_Pd_30_ nanostructured film samples were estimated from SEM images using Image J software [29], where *D* is the nanohole diameter, *W* is the edge-to-edge antidot distance and *C* is the magnetic surface coverage ratio percentage.

Sample	Layer Thickness (nm)	(*D*) (nm)	*W* = *P − D* (nm)	$C=\;1\;-\;\frac{\pi D^{2}}{2\sqrt{3}\;P^{2}}\;100$ (%) [30]
S1	20	69 ± 1	36 ± 2	61 ± 2
S2	40	52 ± 3	53 ± 4	78 ± 3
S3	60	39 ± 3	67 ± 3	87 ± 2
S4	80	18 ± 2	88 ± 2	97 ± 1
S_TF_	-	-	-	100
